# Amenorrhœa of School Girls

**Published:** 1892-05

**Authors:** 


### AMENORRHŒA OF SCHOOL GIRLS.

Dr. T. A. Reamy, (*Cincinnati Lancet-Clinic*), in discussing the amenorrhœa in anæmia, common to school girls, says: (1) She must leave school, and must not even study at home. (2) She must spend several hours each day in the open air, either walking or riding. In winter she must, of course, be warmly clad, but must wear no sheepskins or other chest-protecting pads. Standing in the open air, she must be induced to breathe deeply with the mouth closed; this should be done for at least fifteen or twenty minutes, and be repeated at least twice a day. Nothing that can be done will more rapidly improve the character of her blood. (3) She must sponge her extremities and body each morning on arising from bed. The water must be of the temperature of the room, and she must practice freely with an ordinary towel. (4) See must drink plenty of milk and eat plenty of beef-steak. (5) She must take small doses of iron, combined with some bitter tonic, three times a day. Improvement may be somewhat slow, but if this course is faithfully carried out, a perfect cure will result, and her education may then be finished. If this course or its equivalent be not followed, the cases will go from bad to worse, and finally die from pulmonary tuberculosis.—*Southern Practitioner.*

